# Biomolecular Evidence of Silk from 8,500 Years Ago

**DOI:** 10.1371/journal.pone.0168042

**Published:** 2016-12-12

**Authors:** Yuxuan Gong, Li Li, Decai Gong, Hao Yin, Juzhong Zhang

**Affiliations:** 1 Department of History of Science and Scientific Archaeology, University of Science and Technology of China, Hefei, China; 2 Institute of Cultural Heritage, Shandong University, Jinan, China; 3 Hefei National Laboratory for Physical Sciences at the Microscale, University of Science and Technology of China, Hefei, China; Jilin University, CHINA

## Abstract

Pottery, bone implements, and stone tools are routinely found at Neolithic sites. However, the integrity of textiles or silk is susceptible to degradation, and it is therefore very difficult for such materials to be preserved for 8,000 years. Although previous studies have provided important evidence of the emergence of weaving skills and tools, such as figuline spinning wheels and osseous lamellas with traces of filament winding, there is a lack of direct evidence proving the existence of silk. In this paper, we explored evidence of prehistoric silk fibroin through the analysis of soil samples collected from three tombs at the Neolithic site of Jiahu. Mass spectrometry was employed and integrated with proteomics to characterize the key peptides of silk fibroin. The direct biomolecular evidence reported here showed the existence of prehistoric silk fibroin, which was found in 8,500-year-old tombs. Rough weaving tools and bone needles were also excavated, indicating the possibility that the Jiahu residents may possess the basic weaving and sewing skills in making textile. This finding may advance the study of the history of silk, and the civilization of the Neolithic Age.

## Introduction

Located in the middle of Henan Province, China, Jiahu is one of the most representative early Neolithic Age ruins in central China. Twenty ^14^C dates indicate that the settlement developed over three sub-periods: 9,000 BP to 8,500 BP, 8,500 BP to 8,000 BP, and 8,000 BP to7,500 BP [1–2] (no isotope dates have been obtained directly from the tombs; however, they should fall into these three categories). The site is famous for the discovery of the earliest playable musical instrument (bone flutes) [2], the earliest mixed fermented beverage of rice, honey and fruit [3], the earliest domesticated rice in northern China [4], and possibly the earliest Chinese pictographic writing [5]. The excavated biological remains, including pollen, phytoliths and soil micromorphology, indicate that Jiahu’s warm and humid climate not only favoured the growth of mulberry trees, which feed the silkworm, but also enabled Jiahu inhabitants to settle and develop agriculture [6].

Evidence indicates that the earliest clothes made from animal skin were produced approximately 70,000 years ago or more [7]. Wild flax fibres were made into textiles approximately 30,000 years ago [8]. As a unique material, silk was not used to produce textiles until a much later time; the first use of silk textile is estimated to be only 5,000 years ago [9]. Although previous findings have provided important evidence of silk-making activities, such as figuline spinning wheels and osseous lamellas with traces of filament winding [10], a lack of direct evidence remains a challenge for demonstrating the existence of silk (derived from silkworm) during the Neolithic Age. Silk fibre is a polymer composed of sericin and fibroin, which are two types of proteins. Sericin comprises a series of globular proteins that are unstable and can be damaged rapidly after long-term degradation [11–13]. Fibroin contains highly ordered structural entities that are aggregated by intra- and intermolecular hydrogen bonds. A light chain (approximately 26 kDa) and a heavy chain (approximately 390 kDa) are the two subunits that constitute the fibroin. A heavy chain molecule was identified with twelve domains from the crystalline regions, that containing several Gly-X repeats, with X being Ala, Thr, Ser or Val. A thermodynamically stable structure that ensures the heavy chain’s resistance to water, mild acidity or alkalinity and other degradation factors, was generated due to the strong hydrogen bonds and Van der Waals forces in the crystalline regions [10–17]. Whereas the light chain only connected to the heavy chain by a few disulfide bonds, and is an independent sub-unit that shows less stable properties, for instance more hydrophilic character, higher degradation rate and water uptake ability [17–19]. Hence silk-based cultural relics cannot be easily preserved in their original shape.

Much effort has been made to overcome these difficulties. Credible peptide data obtained in our previous studies provided biomolecular evidence that silk fibroin could be preserved for more than 3,000 years and could be identified in soil with only the trace of textile [20]. We subsequently reported the results of a study whose aim was to distinguish archaeological silk remains from modern silk fibres [21]. In this report, we continue this line of research and focus on identifying the invisible products of silk degradation using mass spectrometry (MS), which is a high-sensitivity, high-accuracy protein identification method. The results showed direct biomolecular evidence of silk fibroin in the soils obtained from 8,500-year-old tombs, which to our knowledge, is the first finding of its kind. Also excavated were rough weaving tools and bone needles, indicating that the detected silk may have been woven or sewn into clothing textiles.

## Methods

### Samples

**Fig 1:**
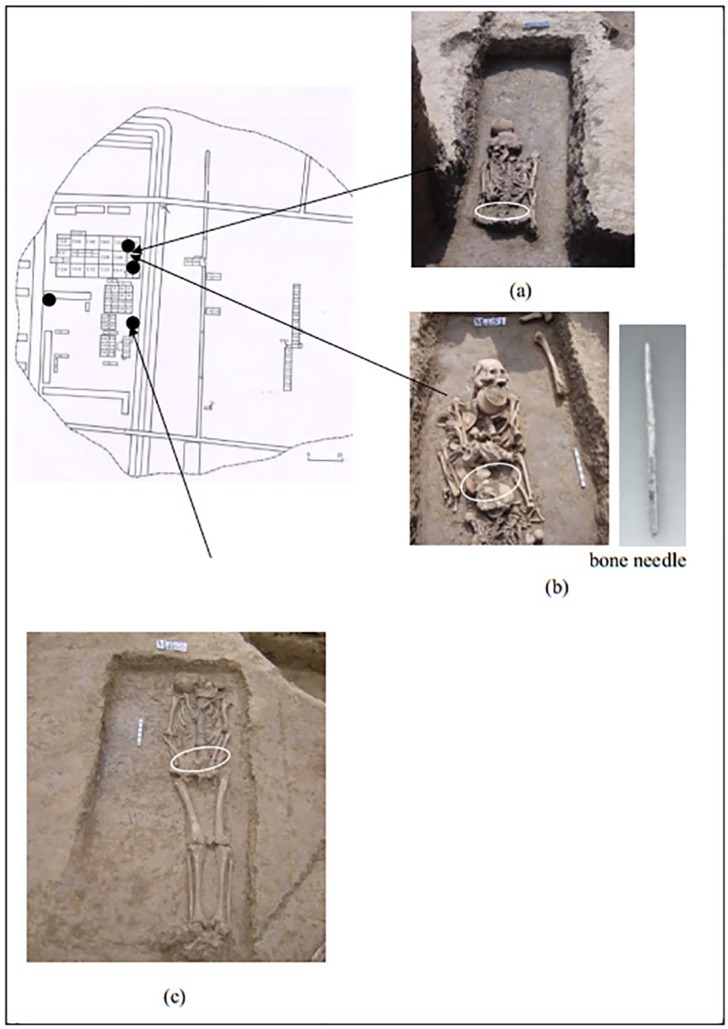
Tombs M436 (a), M451 (b), and M466 (c). The positions where the relic body soil samples were collected are indicated by the arrows and shown in the separate images. The four black dots indicate the locations where the control samples were collected.

As shown in Table 1, the samples were taken from three tombs in Jiahu: M436, M451 and M466. The owner of M436 was a female and appeared to be of a lower class, i.e. she had few funerary objects. M451, a “Mother and Child sharing the grave” tomb type had more burial objects, indicating that its occupants were presumably wealthier. A bone needle was found in this tomb. The owner of M466 was a male, only two burial objects were excavated from this tomb. As shown in Fig 1, the soil samples were collected from beneath the skeletal pelvis, where a semi-closed space is formed that limits the introduction of contaminants. Fabric residue, such as silk protein, from garments worn on the upper and lower body is most likely to be found here. In addition, soil was collected near the tombs for use in control samples (sampling location is marked in Fig 1). All samples were collected simultaneously with the archaeological excavation by experienced investigators to avoid contamination, and were kept sealed in the laboratory prior to the experiments. No permit was required for the described study. The three soil samples (M436, M451, M466) were stored in the Basic Research Center of Heritage Conservation Science, Department of History of Science and Scientific Archaeology, University of Science and Technology of China, which locates at 96 Jinzhai Road, Hefei, P.R. China. These samples are accessible to other interested researchers.

**Table 1:** Information of the samples.

Tomb no.	Tomb size and depth (m)		Dates and descriptions	Figures
M436	Top	Size: 1.1*0.7 Depth: 0.85	Soil samples from the first sub-period of Jiahu (9,000BP-8,500BP)	Fig 1(a)
	Bottom	Size: 1.03*0.7 Depth: 1.33		
M451	Top	Size: 2.4*0.6~0.86 Depth: 1.26		Fig 1(b)
	Bottom	Size: 2.3*0.45~0.85 Depth: 1.5		
M466	Top	Size: 2*0.7 Depth: 0.8	Soil samples from the second sub-period of Jiahu (8,500BP-8,000BP)	Fig 1(c)
	Bottom	Size: 2*0.7 Depth: 0.96		

### Reagents

The chemicals used in the experiments before the digestion (calcium chloride and ethanol) were purchased from Sangon Biotech Co. Ltd, Shanghai, China. Chymotrypsin was supplied by Thermo Fisher Scientific. MeOH and FA were purchased from Sigma—Aldrich (St. Louis, MO).

### Experiments

#### Sample preparation

A soil sample was weighed to 100 g and pulverized, then dissolved in a 150 mL ternary solution (CaCl_2_:H_2_O:C_2_H_5_OH molar ratio 1:8:2) at 95°C for 30 min [22]. The fibroin solution was obtained and placed into 14000 MWCO dialysis filter (Sangon Biotech, Shanghai) to dialyze against 2000 mL deionized water for 48 h with the water renewed every 8 h. After dialysis, the precipitation was removed via membrane separation using syringe filters (pore size 0.45 μM). The concentration was carried out with Amicon Ultra-15 centrifugal filter (Millipore, MWCO = 10 kDa) at 6000 rpm to obtain 100 μL concentrated fibroin solution. The same methods were repeated for each sample.

#### Digestion

A 50 μL concentrated solution of each sample was incubated with 1 μg chymotrypsin at 37°C for 20 h in a new Eppendorf tube (digestion buffers: 10 mM calcium chloride and 500 mM Tris•HCl, pH 8.0). Then the solution was diluted with 0.1% formic acid for MS measurements. The procedure of breaking the disulfide bonds followed by methylation was omitted. As per the explanation in our previous study [21], this does not affect the results.

#### Nano LC-MS/MS

Online reversed-phase (RP) nanoscale capillary liquid chromatography (nano LC) was employed in separating all digested peptide mixtures, and nano-electrospray ionization tandem mass spectrometry (NESI MS/MS) was used in analysing. They were performed on a LC device that connected with an LTQ-Qrbitrap XL mass spectrometer and equipped with a nano-electrospray ion source (Thermo Fisher Scientific). The samples were injected into a 10-cm reversed-phase, fused-silica capillary column (inner diameter 100 μm, packed in-house with a 5-μm Jupiter 300 Å C18, Phenomenex U.S.A.) using an Accela 600 pump (Thermo Fisher Scientific, U.S.A.). The peptides were separated by applying 155-min gradient elution from 10% to 90% solvent B in 80 min. Solvents A and B were HPLC-grade H_2_O with 0.1% FA and LC-MS-grade MeOH respectively. A 10-μL sample solution was loaded at a flow rate of 60 μL/min and eluted at 600 nL/min. The LTQ-Orbitrap XL mass spectrometer was employed in Data-dependent acquisition, positive ion mode was selected. MS survey scans were acquired with a resolution of 60,000 in the Orbitrap, and each scan was recalibrated by an external standard. As many as 5 of the most intense ions per cycle were fragmented and analysed in the linear ion trap. Target ions previously selected for MS/MS were dynamically eliminated for every 90 s. To avoid cross-contamination, blank samples were inserted [20,23,24,25,26,27,28].

#### Database search and data analysis

Automated analysis was performed by Proteome Discoverer 1.2 (Thermo Fisher Scientific) to extract peak lists from the LC-MS/MS data files. The SEQUEST algorithm was run on each of the datasets to match with sequences in both the *B*. *mori*.fasta and *fibroin*.fasta databases from the National Center for Biotechnology Information (NCBI—*B*. *mori*.fasta: release date 1^st^ Oct, 2012; *fibroin*.fasta: release date 1^st^ Oct, 2012), and *uniprot-all*.fasta database from UniProtKB (UniProtKB—*uniprot-all*.fasta: release date 11^th^ May, 2016). For each run, the post-translational modifications of oxidation (M) and deamidation (N/Q) were selected. Each peptide mass tolerance was <3 ppm, and the fragment mass tolerance was <0.8 Da. Except in rare instances, an accepted SEQUEST result was required to have a Δscore of ≥ 0.1 [20,23,25,27]. The detected peptides were then submitted to NCBI online to obtain BLASTp results.

## Results

**Fig 2:**
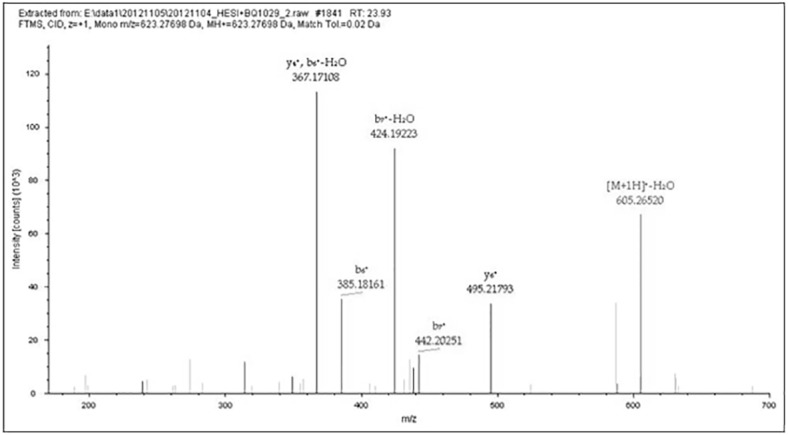
MS/MS results of Peptide GAGAGAGY. This figure shows data-dependent acquisition using the LTQ-Orbitrap XL for the peptide at m/z 623.27698 (GAGAGAGY) in Table 2 (M436 no. 1). The b- and y-type ion fragments of the peptide after CID in MS/MS.

**Fig 3:**
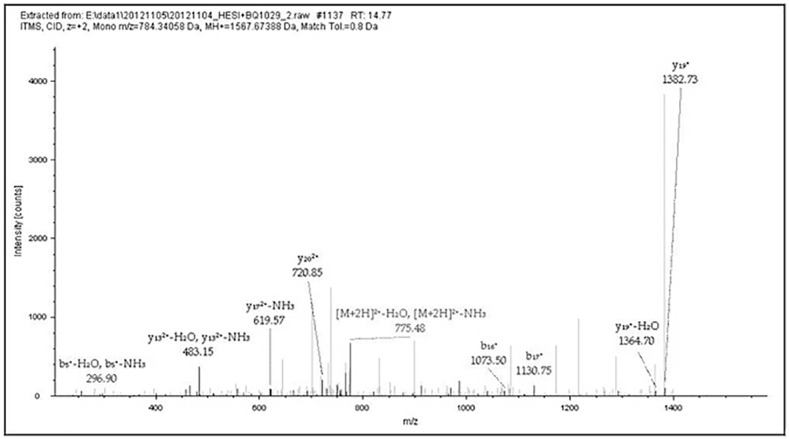
MS/MS results of Peptide GAGAGSGAGSGAGAGSGAGAGY. This figure shows data-dependent acquisition using the LTQ-Orbitrap XL for the peptide at m/z 784.34058 (GAGAGSGAGSGAGAGSGAGAGY) in Table 2 (M436 no. 2). The b- and y-type ion fragments of the peptide after CID in MS/MS.

**Fig 4:**
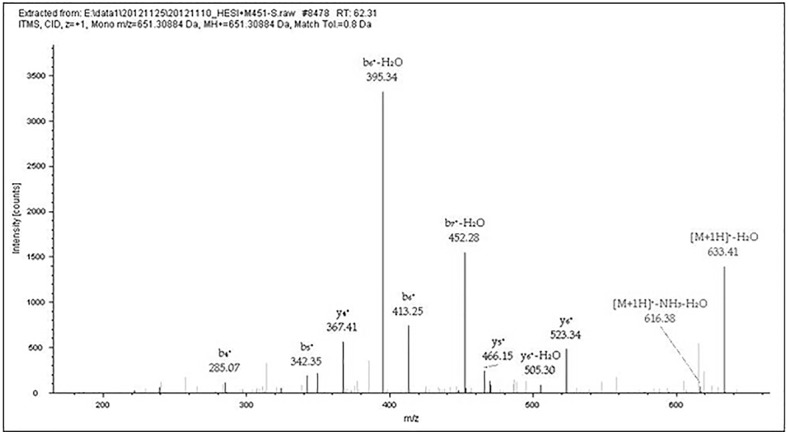
MS/MS results of Peptide GAGVGAGY. This figure shows data-dependent acquisition using the LTQ-Orbitrap XL for the peptide at m/z 651.30884 (GAGVGAGY) in Table 2 (M436 no. 3). The b- and y-type ion fragments of the peptide after CID in MS/MS.

**Fig 5:**
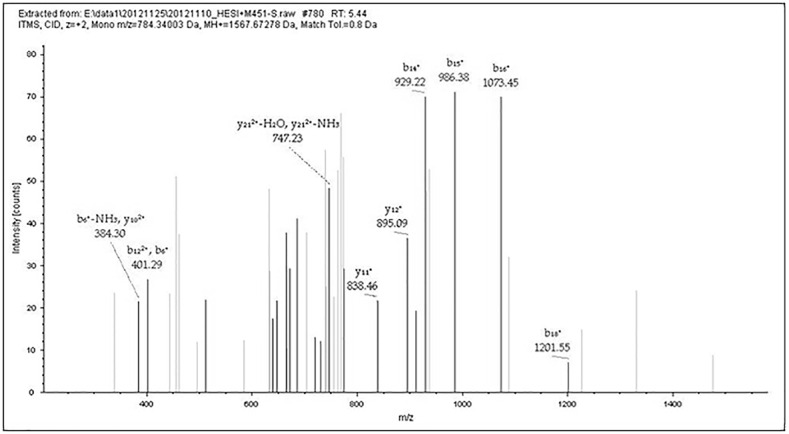
MS/MS results of Peptide GAGAGSGAGSGAGAGSGAGAGY. This figure shows data-dependent acquisition using the LTQ-Orbitrap XL for the peptide at m/z 784.34003 (GAGAGSGAGSGAGAGSGAGAGY) in Table 2 (M451 no. 4). The b- and y-type ion fragments of the peptide after CID in MS/MS.

**Fig 6:**
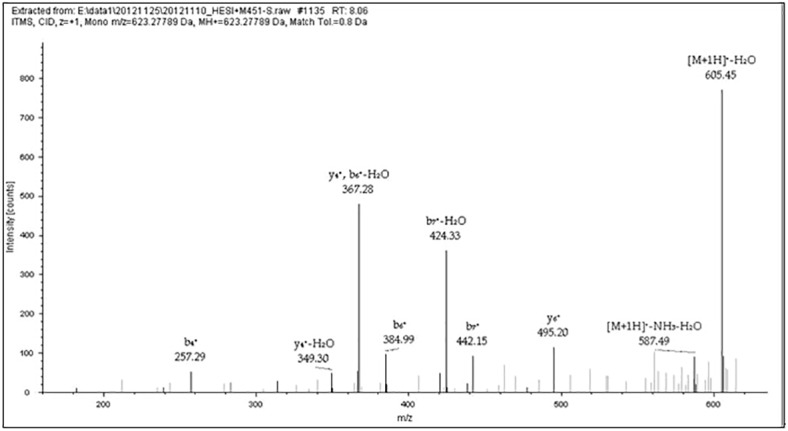
MS/MS results of Peptide GAGAGAGY. This figure shows data-dependent acquisition using the LTQ-Orbitrap XL for the peptide at m/z 623.27789 (GAGAGAGY) in Table 2 (M451 no. 5). The b- and y-type ion fragments of the peptide after CID in MS/MS.

**Fig 7:**
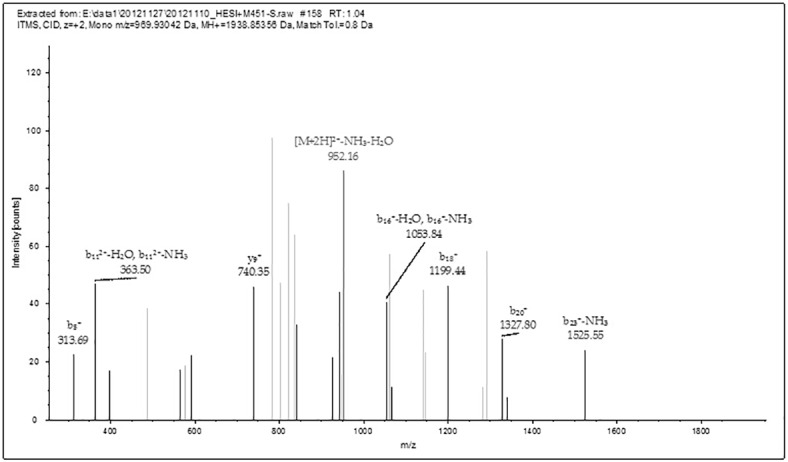
MS/MS results of Peptide GAGAGSGAASGAGAGAGAGAGTGSSGF. This figure shows data-dependent acquisition using the LTQ-Orbitrap XL for the peptide at m/z 969.93207 (GAGAGSGAASGAGAGAGAGAGTGSSGF) in Table 2 (M451 no. 6). The b- and y-type ion fragments of the peptide after CID in MS/MS.

Peptides of silk fibroin were identified in the samples from M436 and M451 by analysing the data-dependent acquisition using nano LC-MS/MS. The sample from M466 and the control samples did not contain silk peptide. Figs 2–7 present the MS/MS spectrograms of the observed peptides listed in Table 2, which all originated from the heavy chain of silk fibroin. Another important common feature of the samples from M436 and M451 is the distinctive restriction fragment, peptide GAGAGAGY at m/z 623.27837 (Theo. mass), which belongs to the crystalline area of the fibroin. The MS/MS data and the accurately deduced sequence results of the GAGAGAGY peptide are presented in Figs 2 and 6. After chymotrypsin digestion (without missed cleavages) of the silk fibroin heavy chain, this octapeptide had as many as 51 copies in one molecule (gi164448672 from NCBI). As a typical peptide with the maximum amount of copies, it was relatively easier to preserve and identify. This peptide was clearly observed in Figs 2 and 6, and was used to deduce the amino acid sequence using the b-type and y-type ion fragments. Other fibroin peptides with similar features were also detected in both samples.

**Table 2:** Peptide sequences of M436 and M451.

Sample	No.	Sequences	Protein	Charge	m/z [Da]	ΔM [ppm]	BLAST results			
							Max score	Coverage	E-value	Identity
M436	1	GAGAGAGY	Fib-H	1	623.27698	-2.28	25.2	100%	379	100%
	2	GAGAGSGAGSGAGAGSGAGAGY	Fib-H	2	784.34058	0.39	60.9	100%	8e-09	100%
M451	3	GAGVGAGY	Fib-H	1	651.30884	-1.33	25.7	100%	728	100%
	4	GAGAGSGAGSGAGAGSGAGAGY	Fib-H	2	784.34003	-0.31	60.9	100%	8e-09	100%
	5	GAGAGAGY	Fib-H	1	623.27789	-0.81	25.2	100%	379	100%
	6	GAGAGSGAASGAGAGAGAGAGTGSSGF	Fib-H	2	969.93207	1.59	64.7	100%	6e-10	100%

## Discussion

Referring to our previous research [20–21], which applied similar methods to ancient samples dated from 403BC-221BC, the results showed the disappearance of the light chain. Most of the detected peptides are characteristic of the crystalline regions of the fibroin heavy chain, and few peptides from amorphous areas were detected. However, abundant information has been obtained from previous research on silk fibroin peptide fragments, including the detection of light and heavy chain fragments in fresh silk fiber [20–21]. The results indicate the decline and disappearance of detected peptide types through degradation. In this study, the data from the M436 and M451 samples shown in Table 2, indicate that most of the surviving peptides belonged to the heavy chain crystalline regions, which is consistent with our previous findings. However, there were also indications that degradation has occurred over a significantly longer time. Few remaining peptides of silk fibroin were identified in the M451 sample while fewer peptides were found in M436. The disappearance of light chain peptides, and the presence of heavy chain peptides in the crystallization regions with one in the amorphous region [17], indicated that the detected remains in the sample of M451 were those of Neolithic silk, and the M436 sample may probably contain silk protein as well.

In addition, no silk peptides were detected in the soil sample from M466, it was therefore considered as a negative sample. It is a possibility that no silk was used in this tomb. However, if silk had ever existed in M466, the result could be interpreted in several ways. The extremely low content is an important factor. The shallow-burial site could have resulted in changes in the burial environment during Jiahu’s second sub-period. These changes may have resulted in adverse preservation conditions for silk, including the introduction of extraneous matter that accelerated the degradation of the silk protein and the migration of organic remains that caused the loss or attenuation of residual silk peptides. Moreover, the negative result also eliminated the possibility that the protein came from other organisms present in the soil.

## Conclusions

The invention of silk was significant not only to ancient China; but to all of Eurasia. As a typical early Neolithic archaeological site in China, Jiahu preserves some of the earliest evidence of human civilization. The results of this paper add silk to this list. The invisible products of the degradation of buried silk were identified in tomb soils using soil proteomics methods. The special peptides of silk protein were detected in two samples, which can be considered new and reliable evidence of the earliest silk fibre in human history. Prehistoric biomolecular evidence of silk in the early Neolithic Age discovered in the tombs of Jiahu; indicates that silk was used more than 8,500 years ago though such burial objects were not ubiquitous. If silk protein or the products of its degradation can survive in different buried environments, biomolecular data could be offered as practical and powerful evidence of the former presence of silk, reducing reliance on circumstantial evidence or guesswork based on archaeological typology. Moreover, these findings are valuable to the study of the history of silk, and advance researchers’ exploration of Neolithic Age civilization.

## Supporting Information

S1 Table The fragment peaks of detected peptides in M436. (DOCX)

S2 Table The fragment peaks of detected peptides in M451. (DOCX)

## References

[1] Zhang JZ. Wuyang Jiahu. 2nd ed Beijing: Science publishing house; 2015.

[2] Zhang JZ, Harbottle G, Wang C, Kong Z. Oldest playable musical instruments found at Jiahu early Neolithic site in China. Nature. 1999; 401(6751): 366–368. 10.1038/43865 16862110

[3] McGovern PE, Zhang JZ, Tang JG, Zhang ZQ, Hall GR, Moreau RA, et al Fermented beverages of pre-and proto-historic China. Proceedings of the National Academy of Sciences of the United States of America. 2004; 101(51): 17593–17598. 10.1073/pnas.0407921102 15590771 PMC539767

[4] Wang X, Sun C, Cai H, Zhang J. Origin of the Chinese cultivated rice (Oryza sativa L.). Chinese science bulletin. 1999; 44(4): 295–304.

[5] Li X, Harbottle G, Zhang J, Wang C. The earliest writing? Sign use in the seventh millennium BC at Jiahu, Henan Province, China. Antiquity. 2003; 77(295): 31–44.

[6] Luo WH. The new evidence of phytolith in regarding to the origin of rice cultivation in Huaihe River Basin. D. Sc. Thesis, University of science and technology of China. 2014.

[7] Balter M. Clothes make the (Hu) man. Science. 2009; 325: 1329. 19745126 10.1126/science.325_1329a

[8] Kvavadze E, Bar-Yosef O, Belfer-Cohen A, Boaretto E, Jakeli N, Matskevich Z, Meshveliani T. 3,000-year-old wild flax fibers. Scicence. 2009; 325: 1359. 10.1126/science.1175404 19745144

[9] Huang NF, Chen JJ. 7,000 year of Chinese silk science and technology. 1st ed Beijing: Chinese textile press; 2002.

[10] Calvert P. Materials science: silk and sequence. Nature. 1998; 393(6683): 309–311.

[11] Ki CS, Park YH, Jin HJ. Silk protein as a fascinating biomedical polymer: Structural fundamentals and applications. Macromolecular Research. 2009; 17(12): 935–942.

[12] Scheibel T. Protein fibers as performance proteins: new technologies and applications. Current opinion in biotechnology. 2005; 16(4): 427–433. 10.1016/j.copbio.2005.05.005 15950453

[13] Vepari C, Kaplan DL. Silk as a biomaterial. Progress in polymer science. 2007; 32(8): 991–1007. 19543442 10.1016/j.progpolymsci.2007.05.013 PMC2699289

[14] Jin HJ, Kaplan DL. Mechanism of silk processing in insects and spiders. Nature. 2003; 424(6952): 1057–1061. 10.1038/nature01809 12944968

[15] Keten S, Xu Z, Ihle B, Buehler MJ. Nanoconfinement controls stiffness, strength and mechanical toughness of [beta]-sheet crystals in silk. Nature materials. 2010; 9(4): 359–367. 10.1038/nmat2704 20228820

[16] Shao Z, Vollrath F. Materials: Surprising strength of silkworm silk. Nature. 2002; 418(6899): 741–741. 12181556 10.1038/418741a

[17] Zhou CZ, Confalonieri F, Jacquet M, Perasso R, Li ZG, Janin J. Silk fibroin: structural implications of a remarkable amino acid sequence. Proteins: Structure, Function, and Bioinformatics. 2001; 44(2): 119–122. 10.1002/prot.1078 11391774

[18] Lu Q, Zhang B, Li MZ, Zuo BQ, Kaplan DL, Huang YL, et al Degradation mechanism and control of silk fibroin. Biomacromolecules. 2011; 12(4): 1080–1086. 10.1021/bm101422j 21361368 PMC3404841

[19] Wadbua P, Promdonkoy B, Maensiri S, Siri S. Different properties of electrospun fibrous scaffolds of separated heavy-chain and light-chain fibroins of Bombyx mori. International journal of biological macromolecules. 2010; 46(5): 493–501. 10.1016/j.ijbiomac.2010.03.007 20338193

[20] Zhu ZY, Chen HF, Li L, Gong DC, Gao X, Y JC, et al Biomass Spectrometry Identification of the Fibre Material in the Pall Imprint Excavated from Grave M1, Peng-state Cemetery, Shanxi, China. Archaeometry. 2013; 56 (4): 681–688.

[21] Li L, Gong YX, Y H, Gong DC. Different types of peptide detected by mass spectrometry among fresh silk and archaeological silk remains for distinguishing modern contamination. Plos One. 2015 7 17. 10.1371/journal.pone.0132827 PMC4505881 26186676

[22] Ajisawa A. Dissolution of silk fibroin with calciumchloride/ethanol aqueous solution. Journal of Sericultural Science of Japan (Japan). 1998; 67 (2): 91–94.

[23] Coligan JE, Dunn BM, Ploegh HL, Speicher DW, Wingfield PT. Current protocols in protein science. New York: John Wiley & Sons; 2004.

[24] Kanerva S, Smolander A, Kitunen V, Ketola R, Kotiaho T. Comparison of extractants and applicability of MALDI—TOF-MS in the analysis of soil proteinaceous material from different types of soil. Organic Geochemistry. 2013; 56: 1–9.

[25] Oonk S, Cappellini E, Collins M. Soil proteomics: An assessment of its potential for archaeological site interpretation. Organic Geochemistry. 2012; 50: 57–67.

[26] Solazzo C, Dyer JM, Deb-Choudhury S, Clerens S, Wyeth P. Proteomic Profiling of the Photo-Oxidation of Silk Fibroin: Implications for Historic Tin-Weighted Silk. Photochemistry and photobiology. 2012; 88(5): 1217–1226. 10.1111/j.1751-1097.2012.01167.x 22554154

[27] Washburn MP, Wolters D, Yates JR. Large-scale analysis of the yeast proteome by multidimensional protein identification technology. Nature biotechnology. 2001; 19(3): 242–247. 10.1038/85686 11231557

[28] Souza G, Godoy L, Mann M. Identification of 491 proteins in the tear fluid proteome reveals a large number of proteases and protease inhibitors. Genome biology. 2006; 7(8): R72.1–R72.11. 16901338 10.1186/gb-2006-7-8-r72 PMC1779605

