# Supplementary material for: Biomolecular Evidence of Silk from 8,500 Years Ago
Source: PLoS One. 2016 Dec 12;11(12):e0168042. doi: 10.1371/journal.pone.0168042 (PMC5152897; doi:10.1371/journal.pone.0168042)
Supplement: S1 Table — (DOCX) [file pone.0168042.s001.docx]

**S1 Table. The fragment peaks of detected peptides in M436**

**Table A the fragment peaks of peptide GAGAGSGAGSGAGAGSGAGAGY**

| **b⁺** | **b²⁺** | **b-H₂O⁺** | **b-H₂O²⁺** | **b-NH₃⁺** | **b-NH₃²⁺** | **Seq.** | **y-H₂O⁺** | **y-H₂O²⁺** | **y-NH₃⁺** | **y-NH₃²⁺** | **y⁺** | **y²⁺** |
| --- | --- | --- | --- | --- | --- | --- | --- | --- | --- | --- | --- | --- |
| 58.02875 | 29.51801 | 40.01818 | 20.51273 | 41.00220 | 21.00474 | G |  |  |  |  |  |  |
| 129.06587 | 65.03657 | 111.05530 | 56.03129 | 112.03932 | 56.52330 | A | 1492.64123 | 746.82425 | 1493.62524 | 747.31626 | 1510.65179 | 755.82953 |
| 186.08734 | 93.54731 | 168.07677 | 84.54202 | 169.06079 | 85.03403 | G | 1421.60411 | 711.30569 | 1422.58812 | 711.79770 | 1439.61467 | 720.31097 |
| 257.12446 | 129.06587 | 239.11389 | 120.06058 | 240.09791 | 120.55259 | A | 1364.58264 | 682.79496 | 1365.56665 | 683.28696 | 1382.59320 | 691.80024 |
| 314.14593 | 157.57660 | 296.13536 | 148.57132 | 297.11938 | 149.06333 | G | 1293.54552 | 647.27640 | 1294.52953 | 647.76840 | 1311.55608 | 656.28168 |
| 401.17796 | 201.09262 | 383.16739 | 192.08733 | 384.15141 | 192.57934 | S | 1236.52405 | 618.76566 | 1237.50806 | 619.25767 | 1254.53461 | 627.77094 |
| 458.19943 | 229.60335 | 440.18886 | 220.59807 | 441.17288 | 221.09008 | G | 1149.49202 | 575.24965 | 1150.47603 | 575.74165 | 1167.50258 | 584.25493 |
| 529.23655 | 265.12191 | 511.22598 | 256.11663 | 512.21000 | 256.60864 | A | 1092.47055 | 546.73891 | 1093.45456 | 547.23092 | 1110.48111 | 555.74419 |
| 586.25802 | 293.63265 | 568.24745 | 284.62736 | 569.23147 | 285.11937 | G | 1021.43343 | 511.22035 | 1022.41744 | 511.71236 | 1039.44399 | 520.22563 |
| 673.29005 | 337.14866 | 655.27948 | 328.14338 | 656.26350 | 328.63539 | S | 964.41196 | 482.70962 | 965.39597 | 483.20162 | 982.42252 | 491.71490 |
| 730.31152 | 365.65940 | 712.30095 | 356.65411 | 713.28497 | 357.14612 | G | 877.37993 | 439.19360 | 878.36394 | 439.68561 | 895.39049 | 448.19888 |
| 801.34864 | 401.17796 | 783.33807 | 392.17267 | 784.32209 | 392.66468 | A | 820.35846 | 410.68287 | 821.34247 | 411.17487 | 838.36902 | 419.68815 |
| 858.37011 | 429.68869 | 840.35954 | 420.68341 | 841.34356 | 421.17542 | G | 749.32134 | 375.16431 | 750.30535 | 375.65631 | 767.33190 | 384.16959 |
| 929.40723 | 465.20725 | 911.39666 | 456.20197 | 912.38068 | 456.69398 | A | 692.29987 | 346.65357 | 693.28388 | 347.14558 | 710.31043 | 355.65885 |
| 986.42870 | 493.71799 | 968.41813 | 484.71270 | 969.40215 | 485.20471 | G | 621.26275 | 311.13501 | 622.24676 | 311.62702 | 639.27331 | 320.14029 |
| 1073.46073 | 537.23400 | 1055.45016 | 528.22872 | 1056.43418 | 528.72073 | S | 564.24128 | 282.62428 | 565.22529 | 283.11628 | 582.25184 | 291.62956 |
| 1130.48220 | 565.74474 | 1112.47163 | 556.73945 | 1113.45565 | 557.23146 | G | 477.20925 | 239.10826 | 478.19326 | 239.60027 | 495.21981 | 248.11354 |
| 1201.51932 | 601.26330 | 1183.50875 | 592.25801 | 1184.49277 | 592.75002 | A | 420.18778 | 210.59753 | 421.17179 | 211.08953 | 438.19834 | 219.60281 |
| 1258.54079 | 629.77403 | 1240.53022 | 620.76875 | 1241.51424 | 621.26076 | G | 349.15066 | 175.07897 | 350.13467 | 175.57097 | 367.16122 | 184.08425 |
| 1329.57791 | 665.29259 | 1311.56734 | 656.28731 | 1312.55136 | 656.77932 | A | 292.12919 | 146.56823 | 293.11320 | 147.06024 | 310.13975 | 155.57351 |
| 1386.59938 | 693.80333 | 1368.58881 | 684.79804 | 1369.57283 | 685.29005 | G | 221.09207 | 111.04967 | 222.07608 | 111.54168 | 239.10263 | 120.05495 |
|  |  |  |  |  |  | Y | 164.07060 | 82.53894 | 165.05461 | 83.03094 | 182.08116 | 91.54422 |

**Table B the fragment peaks of peptide GAGAGAGY**

| **b⁺** | **b-H₂O⁺** | **b-NH₃⁺** | **Seq.** | **y-H₂O⁺** | **y-NH₃⁺** | **y⁺** |
| --- | --- | --- | --- | --- | --- | --- |
| 58.02875 | 40.01818 | 41.00220 | G |  |  |  |
| 129.06587 | 111.05530 | 112.03932 | A | 548.24637 | 549.23038 | 566.25693 |
| 186.08734 | 168.07677 | 169.06079 | G | 477.20925 | 478.19326 | 495.21981 |
| 257.12446 | 239.11389 | 240.09791 | A | 420.18778 | 421.17179 | 438.19834 |
| 314.14593 | 296.13536 | 297.11938 | G | 349.15066 | 350.13467 | 367.16122 |
| 385.18305 | 367.17248 | 368.15650 | A | 292.12919 | 293.11320 | 310.13975 |
| 442.20452 | 424.19395 | 425.17797 | G | 221.09207 | 222.07608 | 239.10263 |
|  |  |  | Y | 164.07060 | 165.05461 | 182.08116 |
